# Ethylene signalling affects susceptibility of tomatoes to *Salmonella*

**DOI:** 10.1111/1751-7915.12130

**Published:** 2014-05-29

**Authors:** Massimiliano Marvasi, Jason T Noel, Andrée S George, Marcelo A Farias, Keith T Jenkins, George Hochmuth, Yimin Xu, Jim J Giovanonni, Max Teplitski

**Affiliations:** 1 Soil and Water Science Department, Genetics Institute, University of Florida-IFASGainesville, FL, 32611, USA; 2 United States Department of Agriculture – Agricultural Research Service and Boyce Thompson Institute for Plant Research, Tower Road, Cornell UniversityIthaca, NY, 14853, USA

## Abstract

Fresh fruits and vegetables are increasingly recognized as important reservoirs of human pathogens, and therefore, significant attention has been directed recently to understanding mechanisms of the interactions between plants and enterics, like *Salmonella*. A screen of tomato cultivars for their susceptibility to *Salmonella* revealed significant differences in the ability of this human pathogen to multiply within fruits; expression of the *Salmonella* genes (*cysB,* *agfB,* *fadH*) involved in the interactions with tomatoes depended on the tomato genotype and maturity stage. Proliferation of *Salmonella* was strongly reduced in the tomato mutants with defects in ethylene synthesis, perception and signal transduction. While mutation in the ripening-related ethylene receptor *Nr* resulted only in a modest reduction in *Salmonella* numbers within tomatoes, strong inhibition of the *Salmonella* proliferation was observed in *rin* and *nor* tomato mutants. RIN and NOR are regulators of ethylene synthesis and ripening. A commercial tomato variety heterozygous for *rin* was less susceptible to *Salmonella* under the greenhouse conditions but not when tested in the field over three production seasons.

## Introduction

From 1998 to 2007 fresh fruits, vegetables, spices and nuts were linked to more outbreaks of human gastroenteritis than either beef, or pork or poultry, with fresh produce sometimes ranked as the riskiest food (Batz *et al*., 2011). Non-typhoidal *Salmonella* has emerged as the most problematic human pathogen associated with fresh produce, nuts and complex foods containing them (deWaal *et al*., 2009; Mandrell, 2009; Batz *et al*., 2011). Despite the apparent importance of vegetables as a vehicle of human gastroenteritis, the approaches for reducing pathogen load in fresh produce could be further improved. At least in part, this lack of food safety solutions is due to the limited understanding of the mechanisms of interactions between enterics and plants.

The ability to colonize plants may be an effective survival strategy for *Salmonella* as it provides a direct route from its excretion in the environment back to its numerous herbivorous and omnivorous hosts (Brandl *et al*., 2013). Under laboratory conditions, multiple routes by which *Salmonella* enters plants' interior were characterized and include invasion of plant lesions, uptake by roots, ingress through hydathodes and stomata, and fruit colonization through the reproductive structures (Guo *et al*., 2001; Cooley *et al*., 2003; Brandl, 2008; Kroupitski *et al*., 2009; Lopez-Velasco *et al*., 2012; Gu *et al*., 2013). The outcomes of plant interactions with *Salmonella* to a significant extent depend on the host: colonization of plant tissues varied not only among plant species but also among genotypes of a given species (Jablasone *et al*., 2005; Klerks *et al*., 2007; Barak *et al*., 2011; Gu *et al*., 2013). Similarly, the plant genotype has an important role in controlling the proliferation of *Escherichia coli* O157:H7 in the lettuce phyllosphere (Quilliam *et al*., 2012). These observations suggest that the interactions between enterics and plants are determined, at least in part, by the host genotype and the associated difference in the biological, physiological and chemical properties of crops, as well as responses to pathogens and endophytes. A better understanding of the host genetic factors involved in restricting (or favouring) proliferation of enterics within plant tissues will be an important step towards devising innovative solutions for improving produce safety.

Even though *Salmonella* is not considered to be a plant pathogen (Barak and Schroeder, 2012; Brandl *et al*., 2013), plants are capable of recognizing it and the associated molecular patterns (Thilmony *et al*., 2006; Schikora *et al*., 2011; Meng *et al*., 2013). Exposure of *Arabidopsis thaliana* to *Salmonella* Typhimurium 14028 and *E. coli* elicited measurable and temporally distinct transcriptomic responses in the plant. One hundred sixty *A. thaliana* genes were commonly upregulated in response to *Salmonella*, *E. coli* K12 and a plant pathogen *Pseudomonas syringae*; however, the magnitude of responses to *Salmonella* or *E. coli* was significantly (50–100×) less than to *P. syringae* (Schikora *et al*., 2011). In another study, inoculation of *A. thaliana* with *E. coli* O157:H7 elicited responses that were distinct from those elicited by the plant pathogen *P. syringae* pv. tomato DC3000 but similar to those elicited by its attenuated mutants (Thilmony *et al*., 2006). The latter included genes belonging to hormone and stress response pathways (Thilmony *et al*., 2006). These observations suggest that plants recognize and respond to enteric pathogens. This conclusion was further supported by the recent characterization of the *Salmonella* Seflg22 as a microbe-associated molecular pattern specifically recognized by plants and leads to the activation of the pathogen-triggered immunity and callose deposition (Garcia *et al*., 2013; Hernandez-Reyes and Schikora, 2013; Meng *et al*., 2013).

There does not yet appear to be a unifying model of a molecular program with which plants respond to human pathogens; however, it is clear that some of the defence and hormone (auxin, ethylene, jasmonic acid) pathways are differentially regulated following interactions of plants with *Salmonella* or *E. coli* O157:H7 (Thilmony *et al*., 2006; Schikora *et al*., 2008; 2011). Salicylic acid-dependent and SA-independent responses have been reported to be involved in the outcome of the interactions of plants with human enteric pathogens [rev. (Brandl *et al*., 2013)]. For example, treatment of *Medicago truncatula* and wheat seedlings with the ethylene precursor 1-aminocyclopropane-1-carboxylic acid (ACC) strongly reduced endophytic populations of *Salmonella* (Iniguez *et al*., 2005). The effect of ACC on the endophytic populations of *Salmonella* depended on the presence of bacterial flagellar genes and Pathogenicity Island I genes encoding functions involved in effector translocation (Iniguez *et al*., 2005).

Further rationale for delineating the involvement of ethylene signalling in produce safety is provided by the fact that some of the commercial tomato varieties contain mutated alleles of ripening genes that are themselves a part of the ethylene signalling. Heterozygousity for *rin* (and, to a lesser extent, *nor*) is often used in tomato breeding programs (Giovannoni, 2007; Garg and Cheema, 2011; Klee and Giovannoni, 2011). NOR is a member of the *NAC*-domain transcription factor family, characterized by the N-terminal DNA-binding domain consisting of five subdomains and a transcriptional regulatory region at the C-terminal (Giovannoni, 2007). A functional LeMADS-RIN, a global regulator of ripening, is required to initiate ethylene biosynthesis in addition to ripening factors that cannot be complemented by exogenous ethylene (Vrebalov *et al*., 2002; Martel *et al*., 2011). While *nor* and *rin* appear to be in the same regulatory pathway (Rohrmann *et al*., 2011; Fujisawa *et al*., 2012), physiological and biochemical changes associated with ripening, accumulation of metabolites, and interactions with pathogens are distinct in the corresponding mutants (Cantu *et al*., 2009; Osorio *et al*., 2011; 2012). Therefore, with this study, we tested proliferation of *Salmonella* in at least a dozen tomato varieties and mutants, including those with defects in ethylene response and ripening processes. Furthermore, expression of the *Salmonella* genes, known to be involved in tomato colonization, was tested in tomato ethylene mutants to determine how and to what extent ethylene signalling affects expression of the known *Salmonella* genes involved in the interactions with tomatoes.

## Results and discussion

### Screen of the existing tomato varieties and mutants for susceptibility to *Salmonella*

**Fig 1:**
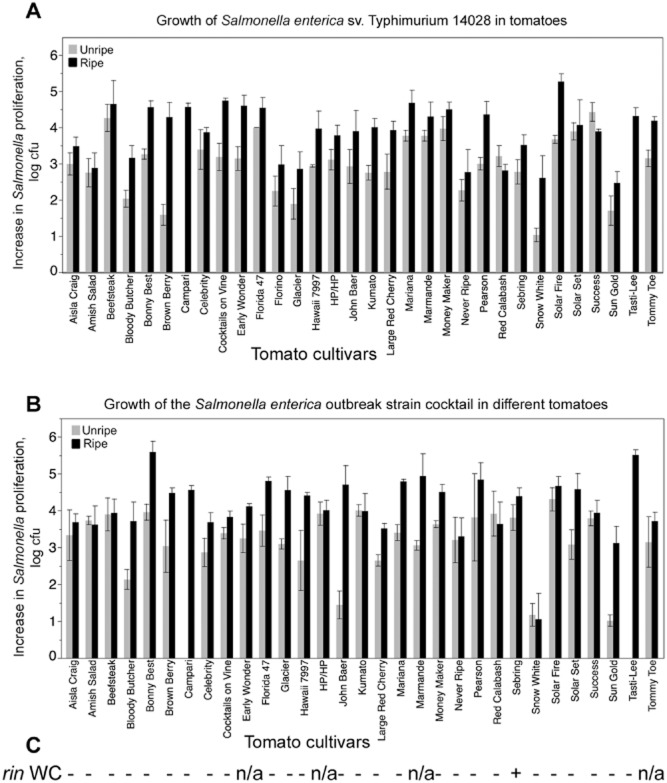
Proliferation of *Salmonella* in ripe and unripe tomatoes of various genotypes. Tomatoes were grown either in the greenhouse or under the field conditions under standard production practices. Red ripe tomatoes of cvs. Campari and Tasti-Lee were purchased in local supermarkets. Tomatoes were inoculated with 100–1000 cells of either *S. enterica* sv. Typhimurium 14028 (A) or a cocktail of the *Salmonella* strains recovered from human outbreaks of illness (B). An increase in proliferation is a log-transformed ratio of the recovered cfu versus inoculum dose. For each variety, at least three technical and three biological tests were done in at least two production seasons. Errors bars are standard errors. (C) A test for heterozygosity revealed that only cv. Sebring is heterozygous for *rin*.

Our screen of 31 tomato varieties was not comprehensive; however, we aimed to include heirloom and commercial varieties with a number of characteristics that could conceivably affect how conducive tomatoes are to *Salmonella*. We have included tomato varieties with known resistances to plant pathogens, including a universally susceptible variety Bonny Best. Because at least one outbreak of salmonellosis was linked to roma-type tomatoes, sampled varieties included beefsteaks, standard-size tomatoes, and roma and cherry types (Fig. 1). Interestingly, cherry tomatoes were generally less conducive to proliferation of *Salmonella* (Supporting Information Fig. S1); however, this observation was not pursued further. In general, it is clear that none of the tested tomato varieties is completely ‘resistant’ to *Salmonella*; however, 10- to 100-fold differences in the populations reached by *Salmonella* within fruits of different cultivars were readily observed (Fig. 1, Supporting Information Table S1).

There were differences in the proliferation of the type strain *Salmonella* Typhimurium 14028 and the cocktail of the outbreak strains (*S. enterica* svs. Javiana ATCC BAA-1593, Montevideo LJH519, Newport C6.3, Braenderup 04E01347, 04E00783, 04E01556) in tomatoes of different varieties, but the differences in the proliferation of the type strain and the *Salmonella* cocktail appear cultivar-specific. This observation is consistent with the reports that under some conditions, strong *Salmonella* serovar-dependent differences in the proliferation within tomatoes, however, these differences were not always reproducible when tomatoes of different varieties were tested (Shi *et al*., 2007; Noel *et al*., 2010a; Marvasi *et al*., 2013a,b).

Strong differences in the *Salmonella* ability to colonize tomatoes at different maturity stages have been reported (Shi *et al*., 2007; Marvasi *et al*., 2013a,b) and are consistent with the observation that ripe fruits are generally more susceptible to opportunistic pathogens (Prusky, 1996). Differences in proliferation of *Salmonella* in mature and immature tomatoes were also observed in this study (Fig. 1). To follow-up on this observation and to attempt to determine the basis underlying this phenomenon, we tested the ability of *Salmonella* to multiply in fruits of the tomatoes with known mutations in ripening-related functions, such as differences in pigmentation, or in ethylene production or perception. For example, the brown colour of fruits of cv. Kumato is due to a *green-flesh* mutation (reduced chlorophyll degradation in ripening fruits (Hu *et al*., 2011). As shown in Fig. 1, populations of *Salmonella* in ripe tomatoes of this variety increased by 10^4^. Proliferation of *Salmonella* Typhimurium ATCC14028 in smaller fruited Brown Berry was reduced in immature tomatoes, but not in mature tomatoes. Even though the nature of the mutation leading to the brown pigmentation in Brown Berry is not defined, these observations collectively suggest that the chlorophyll remaining in the mature fruit tissues is not what is responsible for the reduced proliferation of the pathogen in immature tomatoes. The final cell numbers reached by *Salmonella* in cv. Snow White and Sun Gold (both cherries lacking red pigment when mature) were generally lower than in most tomato varieties and even in some of the red-fruited cherries (e.g. Tommy Toe and Cocktails on the Vine). The deep red colour of mature fruit of cv. Tasti Lee is due to hyperpigmentation (determined by the *HP* mutation) (Wang *et al*., 2008). Even though the cocktail of the outbreak strains was able to increase 5.5 logs in ripe fruit, proliferation of the *Salmonella* Typhimurium 14028 in the ripe fruit of Tasti Lee was an order of magnitude lower. However, when growth of salmonellae in the *HP/HP* mutant and the isogenic wild-type Aisla Craig were compared directly, there were no statistical differences in the proliferation of the pathogen in response to the increased red pigmentation (Fig. 1, Supporting Information Table S1).

Because statistically significant differences in the proliferation of *Salmonella* in tomatoes of different varieties grown under greenhouse conditions were observed (Fig. 1, Supporting Information Fig. S2 and Table S1), field tests were carried out with tomatoes of four cultivars that represented varying levels of ‘resistance’ to *Salmonella* under greenhouse condition (Bonny Best, Florida 47, Sebring and Solar Fire). While tomatoes of the cv. Sebring grown in the greenhouse were less conducive to proliferation of *Salmonella*, a similar trend was not observed in tomatoes of this variety harvested in the field (Supporting Information Fig. S2). Under the field conditions, ripe tomatoes of cv. Florida 47, Bonny Best and Solar Fire were less conducive to *Salmonella* proliferation compared with Sebring (Supporting Information Fig. S2). The mechanism responsible for these observed differences is not yet clear; differences in crop production practices, the diversity of the associated phytomicrobiota are all known to affect the outcomes of interactions between enterics and crops (Gutierrez-Rodriguez *et al*., 2012; Lopez-Velasco *et al*., 2012; Marvasi *et al*., 2013a; Poza-Carrion *et al*., 2013; Williams *et al*., 2013).

### *In vivo* Expression of the *Salmonella* tomato-specific genes

A suite of the *Salmonella* genes differentially regulated in tomatoes has been partially characterized, and their expression was shown to be dependent on the genotype of the plant or fruit's maturity state (Noel *et al*., 2010a; Marvasi *et al*., 2013b). Therefore, with this study, we tested the expression of the *Salmonella* tomato-specific genes within tomatoes of varieties with different levels of ‘susceptibility’ to *Salmonella*, identified in Fig. 1. These reporters included those in *cysB* (a regulator of cysteine biosynthesis and swarming), *agfB* (curli nucleator) and *fadH* (a 2,4-dienoyl-CoA reductase, an iron-sulfur flavoenzyme required for the metabolism of unsaturated fatty acids with double bonds at even carbon positions).

**Fig 2:**
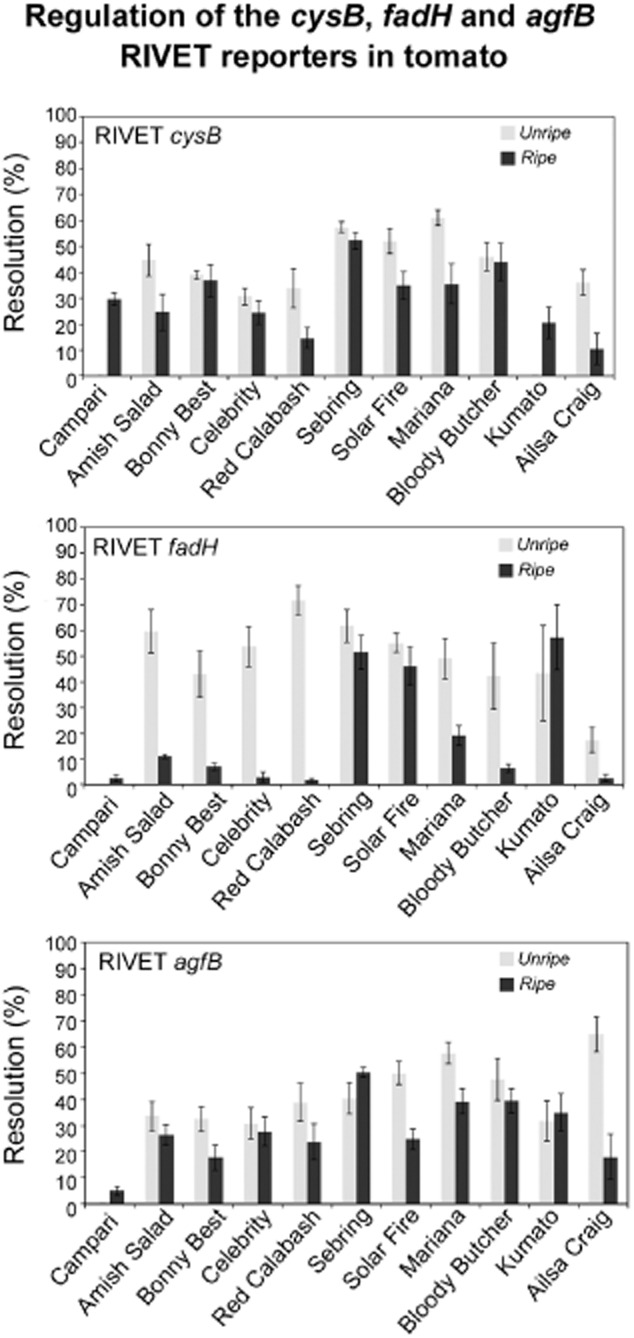
Expression of the *Salmonella* tomato-specific genes in tomatoes of different genotypes. Activity of the Recombinase *in vivo* Expression Technology (RIVET) reporters in the *Salmonella* genes (*cysB*, *fadH*, *agfB*) previously shown to be differentially regulated in tomatoes. Reporters were inoculated into ripe or unripe fruits of the tomatoes of 11 varieties and recovered after a week-long incubation at 24°C. ‘Resolved’ constructs were scored by patching onto tetracycline-containing medium.

Consistent with previous reports, activity of the *cysB* Recombinase *in vivo* Expression Technology (RIVET) reporter was not strongly affected by the maturity of the fruit; however, there were cultivar-level differences in the activity of the reporter (Fig. 2). Because it was previously observed that the expression of *cysB* was highest in the tomato variety with a known resistance to a plant pathogen (Noel *et al*., 2010a) and because CysB regulon is known to be involved in antibiotic resistance (Turnbull and Surette, 2008; 2010), it was hypothesized that the regulation of this gene may correlate with the ability of the tomato variety to sustain proliferation of the pathogen. Consistent with this hypothesis, regression analyses indicate that the expression of the *cysB* reporter in red tomatoes of the tested varieties correlated (*R*^2^ = 0.14038) with the levels reached by the pathogen in red tomatoes; however, no similar trend was observed for green tomatoes (Supporting Information Fig. S3). The highest resolution of the reporter was observed, however, inside red tomatoes of the varieties that tended to sustain higher populations of *Salmonella*. Activity of the *agfB* reporter was generally higher in green tomatoes, compared with red, with the exception of the cultivar Sebring (*rin* heterozygote), in which expression of the *agfB* reporter was higher in red tomatoes (Fig. 2).

Generally consistent with the previous report that the expression of the *fadH* gene was highest in green tomatoes, likely in response to the accumulation of linoleic acid (Noel *et al*., 2010a), resolution of the *fadH* reporter was highest in green tomatoes, with few exceptions. In tomatoes of the cv. Kumato (in which chlorophyll is retained while ripening of the fruit due to a *green-flesh* mutation), mean resolution of the *fadH* reporter was higher in red tomatoes (Fig. 2). In red tomatoes of cvs. Sebring and Solar Fire, resolution of the reporter was higher than in other tomatoes and statistically indistinguishable from the resolution of the reporter in green tomatoes of the same varieties.

### Proliferation of *Salmonella* in tomato ethylene mutants

Because the differences in the pigmentation *per se* do not appear to account for the increased proliferation of the pathogen in red ripe tomatoes compared with green tomatoes and because expression of the *Salmonella* tomato-specific genes in fruit of the variety (Sebring) heterozygous for *rin* was consistently distinct from those with the wild-type ethylene production and detection pathways (Fig. 2), our follow-up experiments focused on determining the contribution of the plant ethylene signalling to the interactions with *Salmonella*.

Under the greenhouse conditions, proliferation of *Salmonella* Typhimurium 14028 and of the cocktail of the outbreak strains in green and red tomatoes of cv. Sebring was generally lower than in fruits of other varieties but also statistically indistinguishable from some of the varieties, which are known to have both wild-type alleles of *Rin* (Fig. 1)*. Salmonella* Typhimurium ATCC 14028 and the cocktail of the outbreak strains grew to significantly lower numbers in the mature tomatoes of the *Never Ripe* mutant compared with the mature fruits of the isogenic parent, cv. Pearson (Fig. 1). The effects of the mutations in the ethylene production and perception are known to depend on the genetic background of tomato (Garg and Cheema, 2011). Therefore, to further standardize experimental conditions, *rin*, *nor* and the *Nr* mutants in the Ailsa Craig background were used for all follow-up experiments.

**Fig 3:**
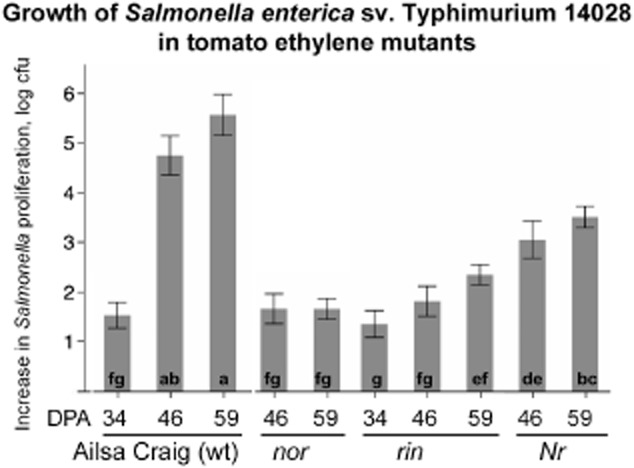
Proliferation of *Salmonella* Typhimurium 14028 in tomato ethylene mutants. Tomato ethylene mutants defective in ethylene perception (*Nr*) or ethylene synthesis and signal transduction (*rin*, *nor*) along with the isogenic parent Ailsa Craig were tested for their ability to support growth of the pathogen. Tomatoes were harvested at 34, 46 or 59 days post-anthesis and 100–1000 cells of *Salmonella* were inoculated into tomatoes and then recovered after a week-long incubation at 24°C. An increase in proliferation is expressed as a log-transformed ratio of the recovered cfu versus the inoculum. Each experiment included at least three technical and three biological replicas; error bars are standard errors. Letters at the bottom of each bar graph represent the Tukey-means separation. Different letters correspond to significantly different means (*P* < 0.05).

When inoculated into developmentally synchronized *rin* and *nor* fruits harvested at 46 or 59 DPA, populations of *Salmonella* increased only 50- to 100-fold. The numbers of the pathogen increased to a similar extent in green (34 dpa) tomatoes of the wild-type Ailsa Craig; however, an increase in the red Ailsa Craig tomatoes (46 or 59 dpa) was 10^5^- to 10^6^-fold. The phenotype of the *nor* tomato mutants was the most severe, and the levels of *Salmonella* within 46 or 59 DPA fruit were similar to those reached by the pathogen in green (34 DPA) fruits of the isogenic parent, Ailsa Craig (Fig. 3). The phenotype of the *rin* mutant was partially relieved at later maturity stages (46 and 59 DPA). In fruits of the *Nr* tomato mutant, which lacks one of the ethylene receptors involved in fruit ripening, *Salmonella* populations increased by approximately 1000-fold, which was higher than in *nor* or *rin* mutants, but significantly less than in the wild type (Fig. 3). Generally, similar trends were observed for the cocktail of the outbreak strains (Supporting Information Fig. S4).

### Effect of ethylene on proliferation of *Salmonella* in tomato mutants

**Fig 4:**
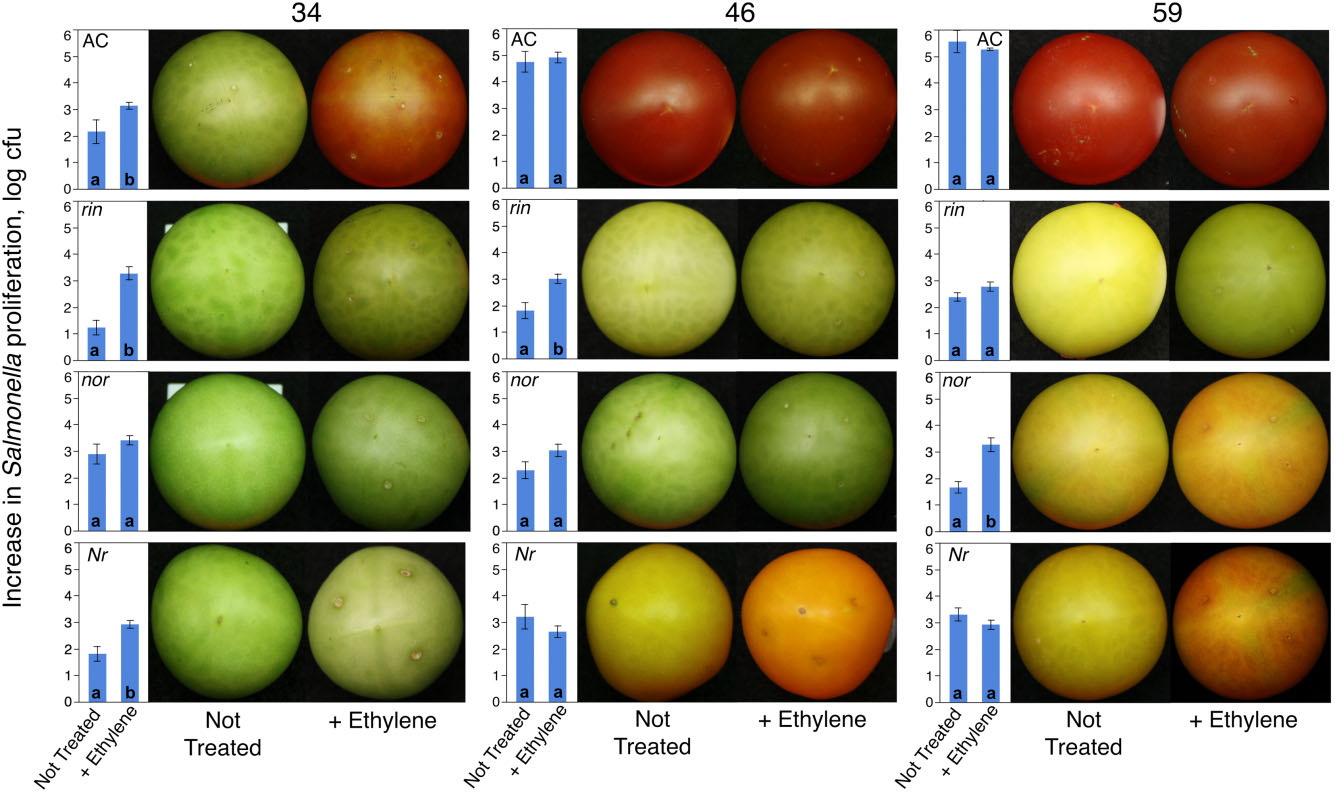
The effect of exogenous ethylene on *Salmonella* proliferation in tomato ethylene mutants. Fruit of the ethylene mutants (*Nr*, *rin*, *nor*) and isogenic parent Ailsa Craig (AC) were harvested at 34, 46 or 59 days post-anthesis, inoculated with *Salmonella* and incubated in a chamber where ethylene was applied to reach 12 ppm every 48 h following a brief venting. As a control, tomatoes were similarly incubated in a chamber-only without supplementation with exogenous ethylene. Tomatoes were sampled after a week-long incubation at 24°C. Blue bars indicate an increase in *Salmonella* Typhimurium 14028 numbers with or without ethylene. Photographs of tomatoes before and after the treatment are also included. Letters within the bars represent results of the pairwise comparisons (*P* < 0.05). Different letters indicate significantly different means.

At 34 dpa, *Salmonella* grew the least in *rin* tomatoes and reached approximately the same final numbers in *nor*, *Nr* and wild-type tomatoes (Fig. 4). Treatment with ethylene resulted in the development of the red colouration of the 34 dpa wild-type tomatoes, and a slight orange colour was apparent in the *Nr* fruits, while *rin* and *nor* mutants remained green. Exposure to ethylene at 34 dpa promoted proliferation of *Salmonella* in the wild type, *rin* and *Nr* mutants but not in *nor* (Fig. 4). It is important to note, however, that the treatment of the wild type, while resulting in the development of the red colour, did not lead to the increase in the *Salmonella* proliferation to the levels observed in red ripe tomatoes.

At 46 dpa, fruits of the wild-type Ailsa Craig were fully red, and *Salmonella* numbers increased by 10^4^–10^5^, which is at least 100-fold higher than in green fruit (34 dpa) and approximately 10-fold higher than in green tomatoes treated with ethylene. Treatment of 46 dpa Ailsa Craig tomatoes with ethylene did not further promote the development of the red colour, nor did it significantly increase proliferation of *Salmonella* (Fig. 4). Treatment of *nor* or *rin* tomatoes did not lead to an increase in the red colour but increased proliferation of the *Salmonella* in the *rin* mutant. Exposure of the 46 dpa *Nr* tomatoes to ethylene increased pigmentation of the fruit but did not lead to an increased proliferation of *Salmonella*.

At 59 dpa, in fruits of the wild-type Ailsa Craig, *Salmonella* further increased by ∼10-fold (compared to 46 dpa); however, treatment with ethylene did not have an impact on further promotion of proliferation of the pathogen (Fig. 4). In *rin* tomatoes, *Salmonella* cell numbers similarly increased by ∼10-fold (compared with 46 dpa), and the treatment with ethylene had only modest effect on the proliferation of the pathogen. In *nor* tomatoes, *Salmonella* populations did not increase compared with 46 dpa, and the treatment with ethylene strongly promoted proliferation of the pathogen in treated fruit. Compared with 46 dpa, there was no further increase in *Salmonella* cell numbers in *Nr* tomatoes at 59 dpa, regardless of the exposure to ethylene (Fig. 4).

These observations suggest that the ability of *Salmonella* to persist in tomatoes depends on the maturity of the fruit and, to some extent, on functionality of the ethylene signalling pathways. The use of the tomato mutants with specific defects in the ethylene synthesis and perception suggests that the ethylene signalling pathways mediated by RIN and NOR (MADS box and SPBP transcriptional factors) are more consequential that those that rely on the ethylene response sensor-like ethylene receptor Nr. In tomatoes, in addition to controlling a common set of transcripts of metabolites, these divergent, but partially overlapping ethylene signalling pathways also control distinct changes in secondary product synthesis, hormone and polyamine metabolism as well as protein turnover (Osorio *et al*., 2011). It is not yet known which of the compounds differentially accumulated in response to ethylene affect proliferation of *Salmonella* in tomatoes. Even though *rin* and *nor* are within the same regulatory pathway (Klee and Giovannoni, 2011; Martel *et al*., 2011; Fujisawa *et al*., 2012; 2013), the corresponding mutations do not have the same effect on the outcomes of tomato interactions with pathogens. For example, *NOR*, but not *RIN*, was required for the control of the susceptibility of tomatoes to *Botrytis cinerea* (Cantu *et al*., 2009).

## Experimental procedures

### Bacterial strains and culture conditions

The following wild-type strains were used in this study: *S. enterica* sv Typhimurium ATCC14028, *Salmonella* Javiana ATCC BAA-1593, *Salmonella* Montevideo LJH519, *Salmonella* Newport C6.3, *Salmonella* Braenderup 04E01347, 04E00783, 04E01556 (the latter six strains were linked to the human outbreaks of salmonellosis resulting from consumption of tomatoes). All strains were maintained as frozen glycerol stocks.

For the tomato infections, bacteria were individually grown overnight at 37°C in Luria Bertani (LB) (Fisher Scientific) broth with shaking at 200 r.p.m. They were then washed twice in PBS (pH 7.0), and the strains from the outbreaks were combined into a six-strain ‘cocktail’ as suggested by the Framework for Evaluation of Microbial Hazards (Harris *et al*., 2012; 2013). These inocula were further diluted in sterile water and 3 μl of the suspension [containing between 10^2^ and 10^3^ colony-forming units (cfu)] were spotted onto three shallow (∼1 mm) wounds in tomato epidermis. Infected tomatoes were incubated at room temperature for a week. Upon completion of the incubation, tomatoes were macerated in an equal volume of 9.8 g l^−1^ of PBS (Fisher Scientific) using a stomacher (Sevard) (200 r.p.m. for 1 min), and the suspensions were plated onto a Xylose Lysine Deoxylate (XLD) agar (Beckton, Dickinson and Company) and incubated at 37–42°C overnight. Proliferation was calculated by dividing the total cfu recovered from each tomato by the total cfu inoculated into each fruit. This provided an accounting for differences in tomato sizes and for the fact that the colonization of a tomato fruit by *Salmonella* is not uniform. The ratios were further subjected to the log_10_ transformation. XLD plates on which there were no *Salmonella* colonies upon completion of the incubation were treated based on the rules of Most Probable Number analysis.

### Reporter assays

RIVET reporters were used for the quantification of *Salmonella* gene expression in tomatoes. Activation of a promoter of interest cloned upstream of the promoterless *tnpR* recombinase gene was determined by scoring the frequency with which TnpR excised an antibiotic resistance cassette cloned in between the ‘res’ sites that are recognized and acted upon by TnpR (Angelichio and Camilli, 2002). For the RIVET assays in tomatoes, *Salmonella* cultures were grown at 37°C overnight in LB supplemented with the appropriate antibiotic(s) (Noel *et al*., 2010a). Bacterial cultures were then pelleted, washed three times in an equal volume of sterile PBS. Approximately 10^2^ cfu (in 3 μl of water) were inoculated onto superficial 1 mm deep wounds on surfaces of unwaxed fruits. At least two technical (individual infections) and three biological (tomatoes from different plants) replications were carried out for each experiment. Unless otherwise stated, infected tomatoes were incubated at 22°C in vented chambers. All RIVET assays were carried out for a week. To harvest samples, 15 × 0.5 mm cores were removed from fruits, homogenized in PBS, and aliquots were then plated onto XLD agar (Oxoid) with appropriate antibiotics. Individual colonies were then patched on LB agar with tetracycline (10 μl ml^−1^) to detect constructs in which TnpR recombinase was active.

### Plant material

For the screen of tomato varieties, tomatoes were grown in the field (two locations/seasons: Citra, FL in Fall 2010 (conventional) and Archer, FL in Spring 2012 (transitional organic) or in the roof-top greenhouse (during the breaks between production seasons). For each variety, field and greenhouse-grown tomatoes were sampled, and the combined data are presented. Seeds were purchased from commercial suppliers. Tomato maturity at harvest was assessed visually. Note that at maturity (corresponding to the USDA chart stages 5 and 6), fruits of Amish Salad, Bonny Best, Celebrity, Red Calabash, Sebring, Solar Fire, Mariana and Bloody Butcher turn red, Brown Berry and Kumato are brown, and Snow White are ivory, while Sun Gold are yellow.

Cultivar Ailsa Craig and lines nearly isogenic for the *rin*, *nor* and *Nr* mutations (Yen *et al*., 1995; Vrebalov *et al*., 2002) were grown in the roof-top greenhouse. To track developmental stages of the fruits, each developing fruit was tagged when it first reached exactly 1 cm in diameter, equal to 7 days post-anthesis (d.p.a.) (Alba *et al*., 2005). In the greenhouse, plants were grown from seed in Miracle-Gro Potting Soil and fertilized biweekly with Miracle-Gro Tomato Plant Food (18-21-21) (Marysville, OH).

### *Rin* genotyping

For genotyping experiments, plants were grown in the greenhouse from seed to approximately four to six true leaf stage. DNA was extracted with a PowerPlant DNA isolation kit (MoBio) according to the manufacturer's instructions. Genotyping for *Rin/rin* alleles was conducted by polymerase chain reaction using primers ATACGATAATGTACAACCCGAAAATG and TCAACTTGAACACACATAAAAAGGAA yielding a 330 bp fragment diagnostic of the wild-type *Rin* allele and primers CTTTCAAACATCATGGCATTGTGGTG and ATATCATTGGCGGAACTTGACGTGAG yielding a 765 bp fragment diagnostic for the mutant *rin* allele.

### Field tomato production

For some experiments, tomatoes (cvs. Bonny Best, Florida 47, Sebring and Solar Fire) were grown in the field over three production seasons in two locations in Florida. Generally, recommended practices for Florida tomato production were used for this research (Olson *et al*., 2012). A cover crop (15 cm tall) of rye (*Secale cereale* L.) was rototilled in preparation for tomato production. Pre-plant fertilizer (13N-2P-10K) was applied at 840 kg ha^−1^ to the bed area and rototilled into the soil prior to bedding and fumigating. The soil at each site was formed into raised beds and fumigated with a mixture of 50% methyl bromide: 50% chloropicrin to control soil-borne pests and weeds. Pre-emergence herbicides were applied carefully to the soil surface in the alleys between beds to control weeds. Black polyethylene mulch was applied to the beds for the spring crops and silver-on-black for the fall. Drip irrigation was applied under the mulch to maintain volumetric water content in the sandy soil (measured by time domain reflectometry) at 8–10% (Munoz-Carpena, 2012). Soluble fertilizer solution (ammonium nitrate and potassium chloride) was injected in 6 biweekly amounts to supplement the pre-plant fertilizers. Total-season N application was 224 kg ha^−1^ N and total-season K application was 210 kg ha^−1^ as K. During the season, fungicides, bactericides and insecticides were applied for pest control as recommended by field scouting and consistent with commercial tomato production practices. For experiments with *Salmonella*, tomatoes were harvested and sorted following normal commercial harvesting practices and brought to the lab for infections with *Salmonella* within 2–24 h of the field harvest.

### Ethylene add-back experiments

Tagged, developmentally synchronized tomatoes (Ailsa Craig wild-type and isogenic *rin/rin*, *nor/nor*, *Nr/Nr* mutants) were harvested at 34, 46 and 59 d.p.a. Tomatoes were inoculated with *Salmonella* Typhimurium 14028 exactly as previously mentioned and then placed inside a 40 × 40 × 20 cm lidded air-tight aquarium, into which 0.39 ml of 100% ethylene were injected with a syringe for a treatment concentration of 12 ppm. Tomatoes were incubated for 1 week, and ethylene injections were repeated every 48 h, following a brief (∼10 min) venting to reduce accumulation of CO_2_. Tomatoes were harvested, and the total proliferation was calculated as described earlier.

## Supporting information

**Fig. S1.** Proliferation of *Salmonella* in cherry-type tomatoes. Proliferation of the type strain of the sv. Typhimurium 14028 and a cocktail of the outbreak strains in cherry-type tomatoes was compared with other varieties (e.g. roma, beefsteaks, etc.). In box plots, rectangles include the lower and upper quartiles, thick lines within the box are medians, and whiskers indicate the degree of dispersion of the data. Outlier data are shown as dots. Letters above box plots represent results of the pairwise comparisons (*P* < 0.05). Different letters correspond to significantly different means.

**Fig. S2.** Susceptibility of the tomatoes to *Salmonella* as affected by cultivar and method of production (greenhouse versus field). Tomatoes Bonny Best, Florida 47, Sebring and Solar Fire were grown in the greenhouse over at least two production seasons and in the field, as indicated in Experimental procedures. In the field, tomatoes were produced in three production seasons in two geographical locations in Florida. Harvested tomatoes were brought into the lab and inoculated either with *Salmonella* Typhimurium 14028 or with the outbreak strain cocktail. An increase in the *Salmonella* numbers was measured after a week. Only green (immature) and red (mature) tomatoes are included in the assessment. Tomatoes that ripened during the experiment were excluded from the comparison. In box plots, rectangles include the lower and upper quartiles, thick lines within the box are medians, and whiskers indicate the degree of dispersion of the data. Outliers are shown as dots. Letters above box plots represent Tukey means separation. Different letters correspond to significantly different means (*P* < 0.05).

**Fig. S3.** Correlation between expression of *cysB*, *fadH*, *agfB* and the overall proliferation of *Salmonella* in tomatoes. Resolution of the RIVET reporters in *cysB*, *fadH* and *agfB* (see Fig. 2) was correlated with the overall phenotype (Fig. 1). Red squares are mature (red) tomatoes, and green circles are immature (green) tomatoes. Continuous and dotted lines represent the linear regression for mature and immature tomatoes respectively. The coefficients of determination (*R*^2^) for the linear regressions were *cysB* 0.14038, 0.01494; *fadH* 0.13372, 0.03229; and *agfB* 0.00512, 0.00261 for mature and immature tomatoes respectively.

**Fig. S4.** Proliferation of the cocktail of *Salmonella* outbreak strains in tomato ethylene mutants. Tomato ethylene mutants defective in ethylene perception (*Nr*) or ethylene synthesis and signal transduction (*rin*, *nor*) along with the isogenic parent Ailsa Craig were tested for their ability to support growth of the pathogen. Tomatoes were harvested at 34, 46 or 59 days post-anthesis and 100–1000 cells of *Salmonella* were inoculated into tomatoes and then recovered after a week-long incubation. An increase in proliferation is expressed as a log-transformed ratio of the recovered cfu versus the inoculum. Each experiment included at least three technical and three biological replicas; error bars are standard errors. Letters at the bottom of each bar graph represent the Tukey-means separation. Different letters correspond to significantly different means (*P* < 0.05).

**Table S1.** Proliferaton of *Salmonella enterica* in tomatoes of different varieties: Tukey–Kramer means separation.
