# Supplementary figures and images for: Ethylene signalling affects susceptibility of tomatoes to Salmonella
Source: Microb Biotechnol. 2014 May 29;7(6):545–55. doi: 10.1111/1751-7915.12130 (PMC4265073; doi:10.1111/1751-7915.12130)

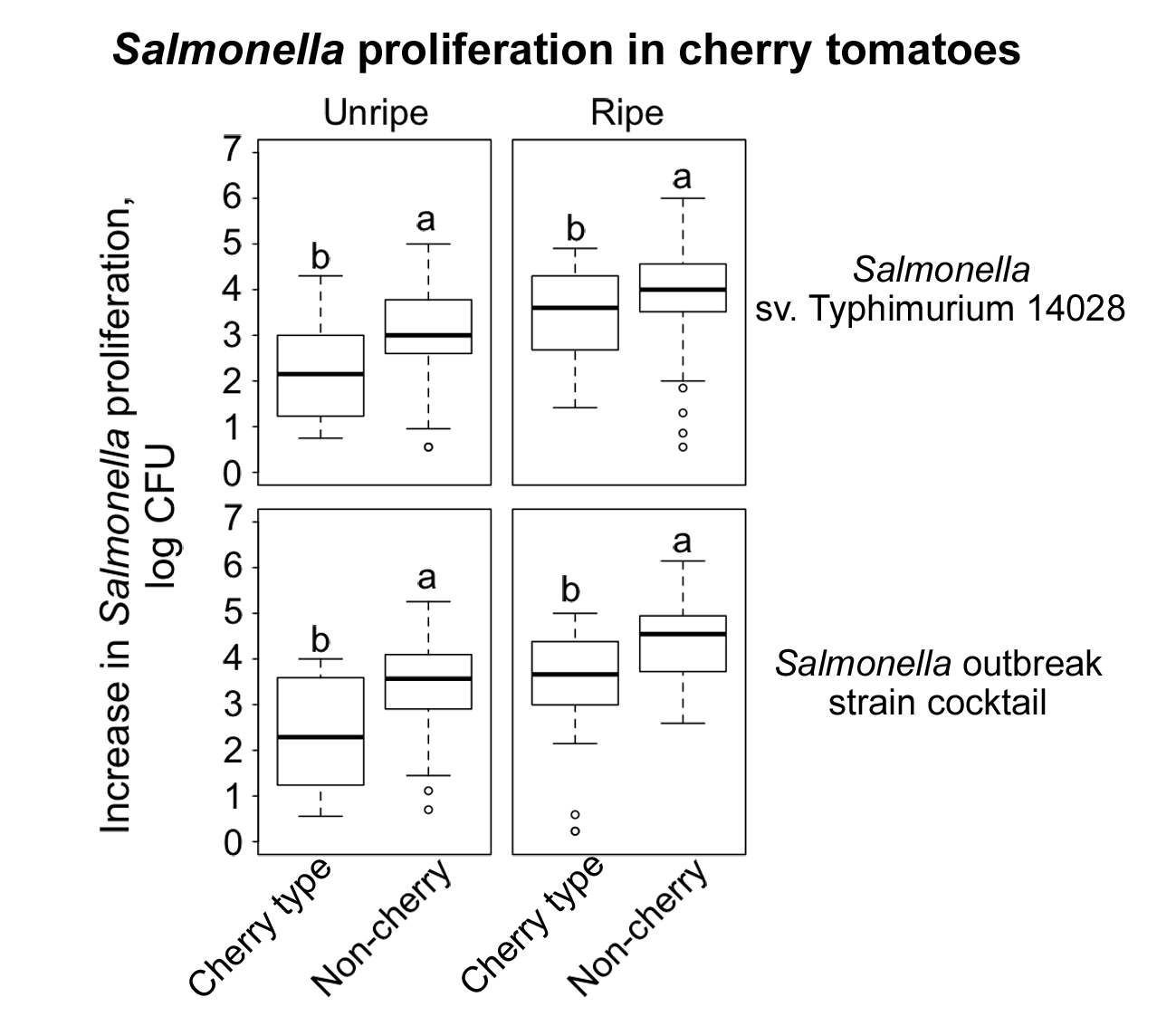

Supplement: Supplementary file 1 — Fig. S1. Proliferation of Salmonella in cherry-type tomatoes. Proliferation of the type strain of the sv. Typhimurium 14028 and a cocktail of the outbreak strains in cherry-type tomatoes was compared with other varieties (e.g. roma, beefsteaks, etc.). In box plots, rectangles include the lower and upper quartiles, thick lines within the box are medians, and whiskers indicate the degree of dispersion of the data. Outlier data are shown as dots. Letters above box plots represent results of the pairwise comparisons (P < 0.05). Different letters correspond to significantly different means. [file mbt20007-0545-sd1.tif]

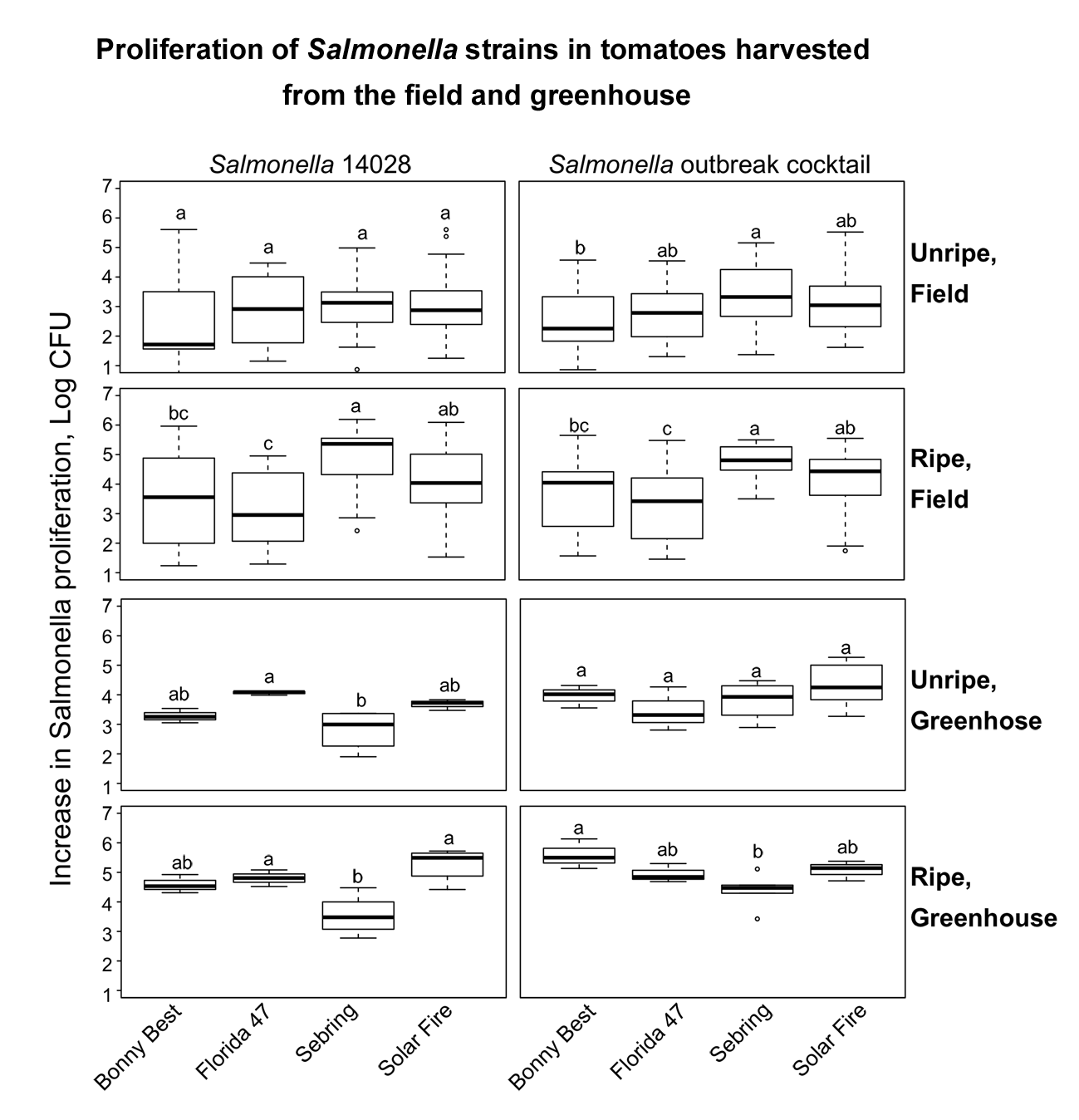

Supplement: Supplementary file 2 — Fig. S2. Susceptibility of the tomatoes to Salmonella as affected by cultivar and method of production (greenhouse versus field). Tomatoes Bonny Best, Florida 47, Sebring and Solar Fire were grown in the greenhouse over at least two production seasons and in the field, as indicated in Experimental procedures. In the field, tomatoes were produced in three production seasons in two geographical locations in Florida. Harvested tomatoes were brought into the lab and inoculated either with Salmonella Typhimurium 14028 or with the outbreak strain cocktail. An increase in the Salmonella numbers was measured after a week. Only green (immature) and red (mature) tomatoes are included in the assessment. Tomatoes that ripened during the experiment were excluded from the comparison. In box plots, rectangles include the lower and upper quartiles, thick lines within the box are medians, and whiskers indicate the degree of dispersion of the data. Outliers are shown as dots. Letters above box plots represent Tukey means separation. Different letters correspond to significantly different means (P < 0.05). [file mbt20007-0545-sd2.tif]

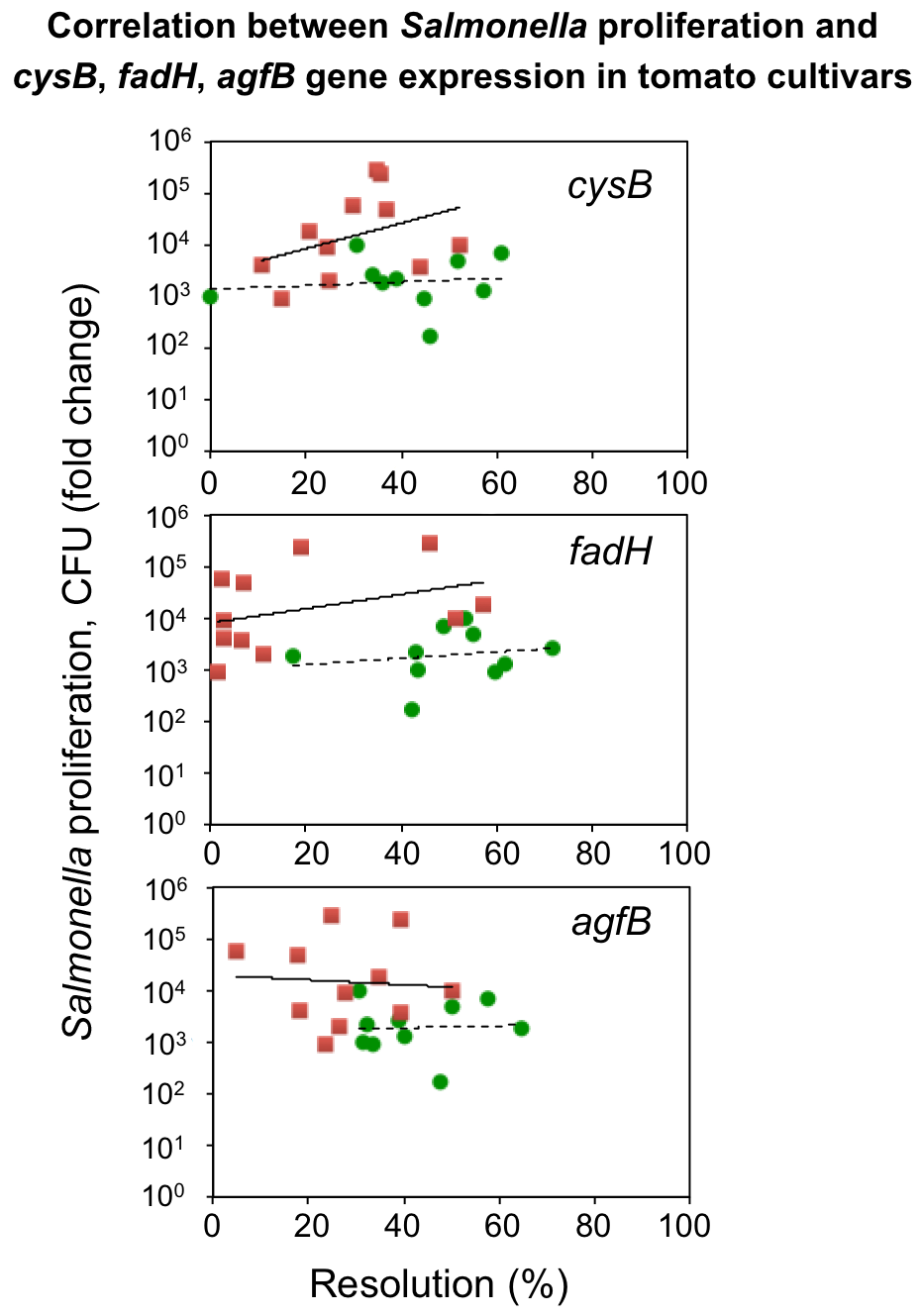

Supplement: Supplementary file 3 — Fig. S3. Correlation between expression of cysB, fadH, agfB and the overall proliferation of Salmonella in tomatoes. Resolution of the RIVET reporters in cysB, fadH and agfB (see Fig. 2) was correlated with the overall phenotype (Fig. 1). Red squares are mature (red) tomatoes, and green circles are immature (green) tomatoes. Continuous and dotted lines represent the linear regression for mature and immature tomatoes respectively. The coefficients of determination (R2) for the linear regressions were cysB 0.14038, 0.01494; fadH 0.13372, 0.03229; and agfB 0.00512, 0.00261 for mature and immature tomatoes respectively. [file mbt20007-0545-sd3.tif]

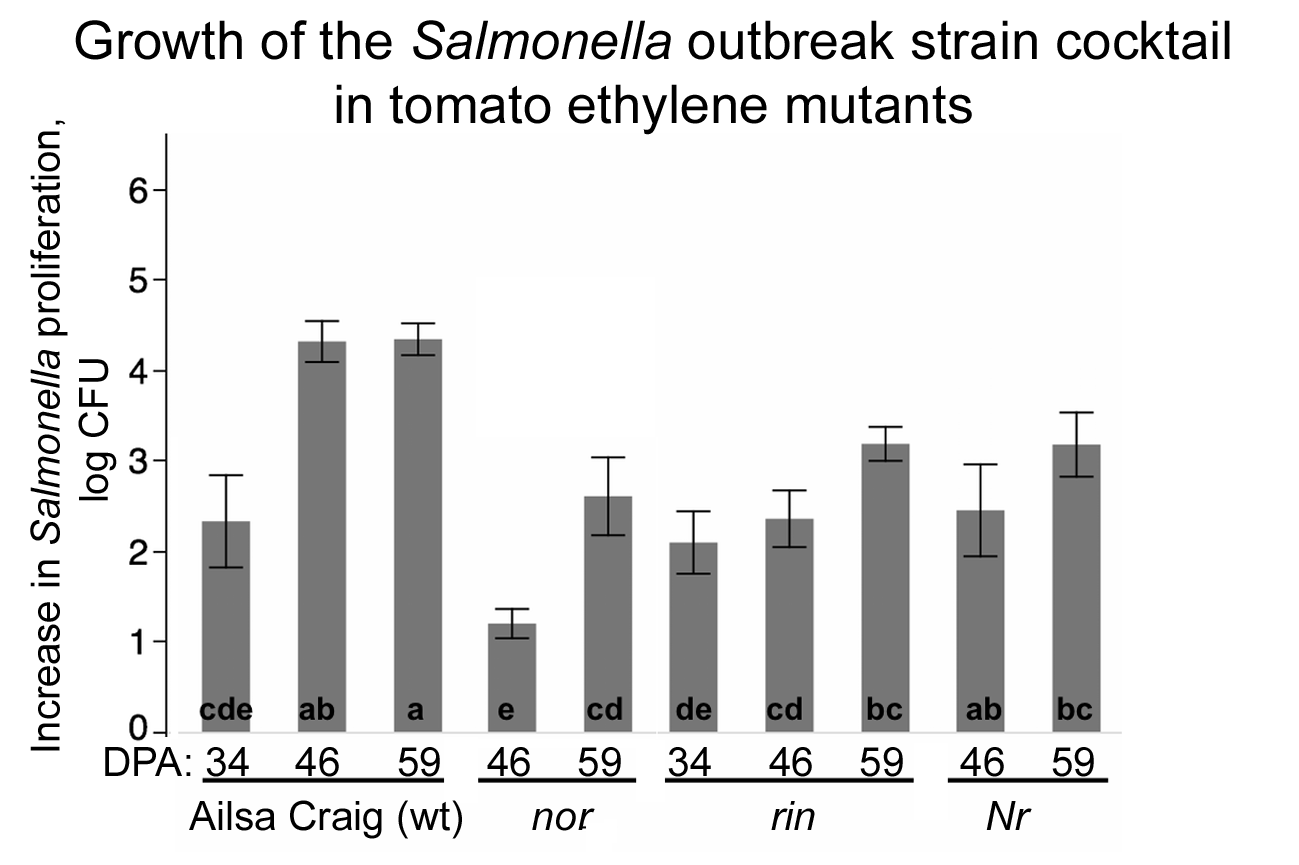

Supplement: Supplementary file 4 — Fig. S4. Proliferation of the cocktail of Salmonella outbreak strains in tomato ethylene mutants. Tomato ethylene mutants defective in ethylene perception (Nr) or ethylene synthesis and signal transduction (rin, nor) along with the isogenic parent Ailsa Craig were tested for their ability to support growth of the pathogen. Tomatoes were harvested at 34, 46 or 59 days post-anthesis and 100–1000 cells of Salmonella were inoculated into tomatoes and then recovered after a week-long incubation. An increase in proliferation is expressed as a log-transformed ratio of the recovered cfu versus the inoculum. Each experiment included at least three technical and three biological replicas; error bars are standard errors. Letters at the bottom of each bar graph represent the Tukey-means separation. Different letters correspond to significantly different means (P < 0.05). [file mbt20007-0545-sd4.tif]

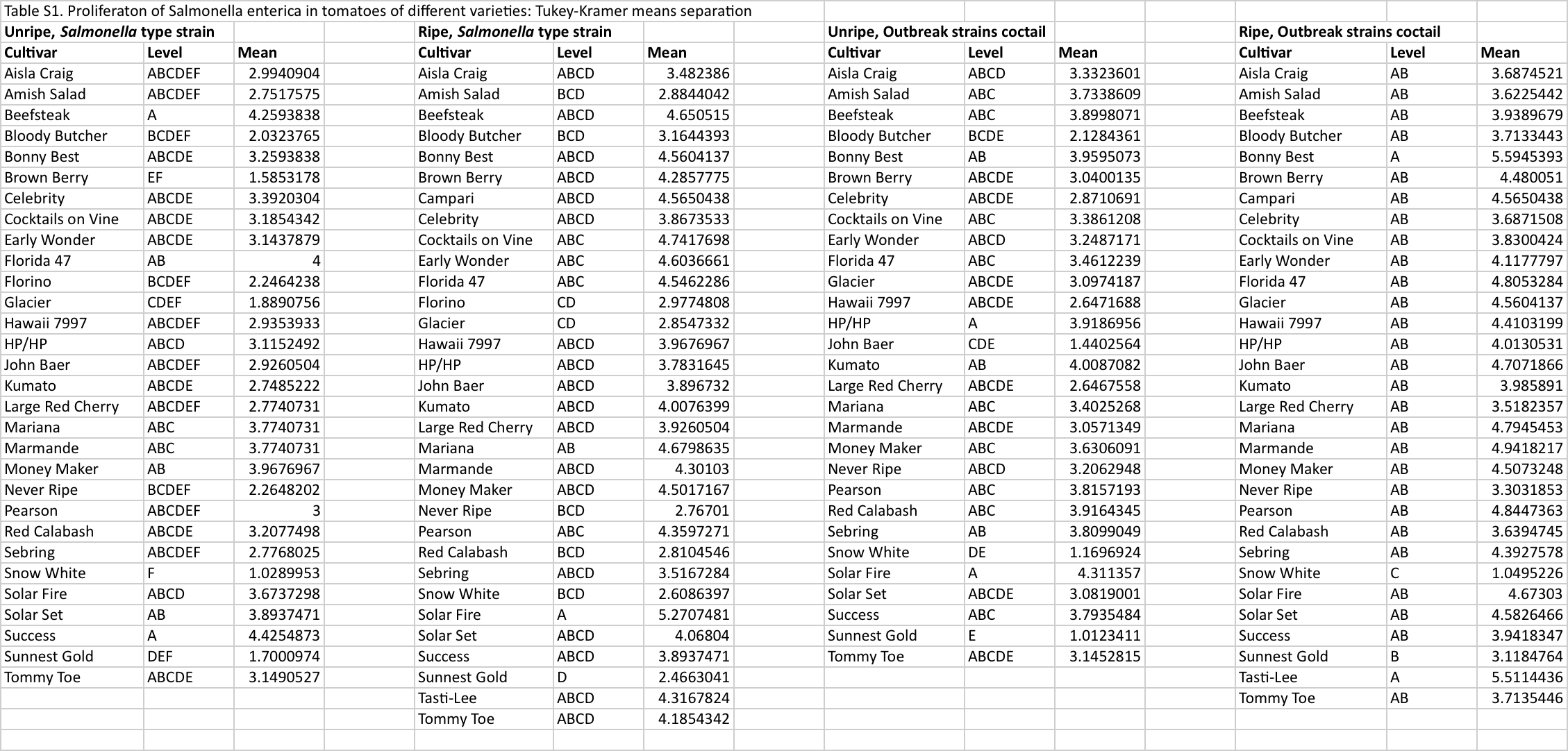

Supplement: Supplementary file 5 — Table S1. Proliferaton of Salmonella enterica in tomatoes of different varieties: Tukey–Kramer means separation. [file mbt20007-0545-sd5.docx]
